# Rectal forceps biopsy procedure in cystic fibrosis: technical aspects and patients perspective for clinical trials feasibility

**DOI:** 10.1186/1471-230X-13-91

**Published:** 2013-05-20

**Authors:** Maria F Servidoni, Marisa Sousa, Adriana M Vinagre, Silvia R Cardoso, Maria A Ribeiro, Luciana R Meirelles, Rita B de Carvalho, Karl Kunzelmann, Antônio F Ribeiro, José D Ribeiro, Margarida D Amaral

**Affiliations:** 1 Gastrocentro - Endoscopy Unit - State University of Campinas (Unicamp) - Cidade Universitária Zeferino Vaz - Barão Geraldo, Campinas, SP 13083-872, Brazil; 2 Post-graduate Course, Pediatrics Department, State University of Campinas (Unicamp) - Cidade Universitária Zeferino Vaz - Barão Geraldo, Campinas, SP 13083-872, Brazil; 3 University of Lisboa - Faculty of Sciences, BioFIG - Centre for Biodiversity, Functional and Integrative Genomics, Campo Grande, 1749-016, Lisbon, Portugal; 4 Department of Genetics, National Institute of Health – Av. Padre Cruz, 1649-016, Lisbon, Portugal; 5 Faculty of Medical Sciences - State University of Campinas (Unicamp) - Cidade Universitária Zeferino Vaz - Barão Geraldo, Campinas, SP 13083-872, Brazil; 6 Endoscopy Unit – University Hospital of Campinas, Campinas, Brazil; 7 CIPED - Research Center in Pediatrics - State University of Campinas (Unicamp) - Cidade Universitária Zeferino Vaz - Barão Geraldo, Campinas, SP 13083-872, Brazil; 8 Pathological Anatomy Department, University Hospital of Campinas (Unicamp) - Cidade Universitária Zeferino Vaz - Barão Geraldo, Campinas, SP 13083-872, Brazil; 9 Institut für Physiologie - Universität Regensburg, Universitat Strasse 31, D-93053, Regensburg, Germany; 10 Edifício C8, Departamento de Química e Bioquímica, Faculdade de Ciências da Universidade de Lisboa, Campo Grande, 1749-016, Lisbon, Portugal

**Keywords:** Quality control, Patient comfort, Forceps, Outcome measures clinical trial, Rectal biopsy

## Abstract

**Background:**

Measurements of CFTR function in rectal biopsies *ex vivo* have been used for diagnosis and prognosis of Cystic Fibrosis (CF) disease. Here, we aimed to evaluate this procedure regarding: *i)* viability of the rectal specimens obtained by biopsy forceps for *ex vivo* bioelectrical and biochemical laboratory analyses; and *ii)* overall assessment (comfort, invasiveness, pain, sedation requirement, etc.) of the rectal forceps biopsy procedure from the patients perspective to assess its feasibility as an outcome measure in clinical trials.

**Methods:**

We compared three bowel preparation solutions (NaCl 0.9%, glycerol 12%, mannitol), and two biopsy forceps (standard and jumbo) in 580 rectal specimens from 132 individuals (CF and non-CF). Assessment of the overall rectal biopsy procedure (obtained by biopsy forceps) by patients was carried out by telephone surveys to 75 individuals who underwent the sigmoidoscopy procedure.

**Results:**

Integrity and friability of the tissue specimens correlate with their transepithelial resistance (r = −0.438 and −0.305, respectively) and are influenced by the bowel preparation solution and biopsy forceps used, being NaCl and jumbo forceps the most compatible methods with the electrophysiological analysis. The great majority of the individuals (76%) did not report major discomfort due to the short procedure time (max 15 min) and considered it relatively painless (79%). Importantly, most (88%) accept repeating it at least for one more time and 53% for more than 4 times.

**Conclusions:**

Obtaining rectal biopsies with a flexible endoscope and jumbo forceps after bowel preparation with NaCl solution is a safe procedure that can be adopted for both adults and children of any age, yielding viable specimens for CFTR bioelectrical/biochemical analyses. The procedure is well tolerated by patients, demonstrating its feasibility as an outcome measure in clinical trials.

## Background

Cystic Fibrosis (CF), the most common life shortening autosomal recessive disease of Caucasian populations, is caused by mutations in the CF transmembrane conductance regulator (CFTR) gene encoding a chloride (Cl^-^) channel expressed at the apical membrane of epithelial cells, a major regulator of salt and water transport in epithelia [1]. CF is dominated by respiratory disease but other organs are also affected including the pancreas, intestine and sweat gland as well as male reproductive tract [2].

Although the clinical diagnosis of classic (severe) forms of CF is straightforward, for other patients there is wide variability in the clinical presentation and organ involvement, thus making the CF diagnosis more challenging [3-7]. Moreover, increasing numbers of asymptomatic patients are currently identified through extended programs of CF newborn screening [4,8-11].

One of the most useful and sensitive laboratory parameters used for the diagnosis and prognosis of CF, is *ex vivo* assessment of CFTR-mediated Cl^-^ secretion channel in freshly collected rectal biopsies [8,9,12-19]. Moreover, ongoing clinical trials of novel therapeutic CFTR-modulators require improved and robust biomarkers to adequately assess their *in vivo* efficacy on CFTR. Indeed, there is also great potential to exploit this method to pre-clinically assess compound efficacy directly on human tissues *ex vivo*, as we previously showed [20] or as a biomarker in clinical trials of novel CFTR-modulators [21-23]. Moreover, it may even be used to evaluate patient/CFTR genotype responsiveness to a drug through a personalized-medicine approach.

For such disseminated usage of this method, standardized operational procedures (SOP) for bowel preparation and biopsing are essential to ensure good tissue viability for the quantitative assessment of the bioelectric parameters [13]. Moreover, since the procedure involves biopsing, a somewhat invasive procedure possibly triggering psychological rejection, there should be clear information on how it is perceived from the patients’ perspective (comfort, invasiveness, pain, sedation requirement, etc.) to obtain an overall assessment of the method.

Our two-fold aim of the present study was: a) to evaluate the technical procedure regarding the quality for bioelectrical/biochemical laboratory analyses of 580 rectal specimens from 132 individuals (CF and non-CF), namely by comparing different bowel preparations and different biopsy forceps sizes as well as regarding the safety of the procedure to the patient; and b) to determine individuals’ assessment regarding the rectal biopsy procedure feasibility to be possibly used as an outcome measure in clinical trials, as successfully described in other studies [24,25].

Our results demonstrate that best tissue viability for Ussing chamber measurements results after bowel preparation with isotonic solution (NaCl 0.9%) and usage of jumbo (*vs* standard size) biopsy forceps allowing collection of larger specimens without disrupting tissue integrity. Obtaining rectal biopsies with a flexible endoscope and jumbo forceps is also shown here to be a safe procedure for use in both children and adults (age range was 6 months to 52 years). Data collected on patient’s comfort show that the great majority of individuals (76%) did not report major discomfort due to the short procedure time (max 15 min) and considered it relatively painless (79%), regardless of sedation. Moreover, most individuals accept repeating the procedure for at least one more time (88%) and 53% for more than 4 times, supporting the feasibility of the current approach as an outcome measure in clinical trials.

## Methods

### Subjects

**Figure 1:**
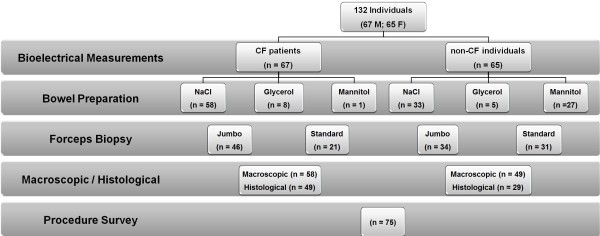
**Flow-chart of the technical biopsing aspects assessed in the present study.** Bioelectrical measurements were performed for rectal biopsies (n = 580) from all the individuals enrolled in the study (n = 132) to assess tissue viability [14]. Bowel preparation included enemas of either NaCl 0.9%, glycerol 12% (v/v) or oral mannitol 20% (w/v) solutions. Two different biopsy forceps were tested, namely jumbo (3.4 mm Ø) and standard (2.5 mm Ø), independently of bowel preparation. Macroscopic and histologic evaluation of rectal biopsies was achieved for 107 and 78 individuals, respectively. Patient assessment surveys were carried out for 75 individuals undergoing sigmoidoscopy with rectal biopsy collection by biopsy forceps, divided into 4 age groups, namely (yrs): 0–9; 10–9; 20–29; ≥30.

Access to human tissues used in this study received approval from the Research Ethics Committee of the Faculty of Medical Sciences, State University of Campinas (Unicamp, ref. 503/2007), in accordance with the Helsinki Declaration of the World Medical Association. Signed informed consent was obtained from all individuals (or parents/tutors, for those <18 yrs). Altogether, 580 freshly excised rectal biopsies were analysed at Unicamp from 132 individuals, including CF patients (n = 67) with previously established diagnosis [14] (genotypes in Additional file 1: Table S6 and in Sousa et al [14]) and age-matched non-CF individuals (n = 65) undergoing biopsing for other clinical reasons and agreeing to participate in the study (Figure 1). The number of biopsies found suitable for quantitative bioelectrical measurements was 404 (i.e., ~70%). Assessment of the overall rectal biopsy procedure by patients was carried out by telephone surveys to individuals (n = 75) who underwent the sigmoidoscopy/rectal forceps biopsy procedure (Figure 1).

### Bowel preparation

Bowel preparation was done on-site (~30 min prior to sigmoidoscopy) by applying an enema of *i)* saline solution (0.9% NaCl, total volume: 0.25-1 L); or *ii)* glycerinated solution (12% glycerol in distilled water, total volume: 0.25-1 L), for individuals undergoing sigmoidoscopy; or *iii)* oral mannitol solution (20%, total volume: 0.5 L on the previous day) for individuals undergoing colonoscopy (Figure 1). Satisfactory bowel cleaning was achieved in 99.24% (131/132) of cases. At our endoscopy unit, oral mannitol is used only when a complete cleaning of the bowel is required; while the routine solutions used for sigmoidoscopy are usually glycerinated solution or saline solution. These solutions are cheap and offer low risk of dehydration for the patient (especially for people that live in very hot climates, like Brazil). Also in the case of rectal biopsies, only the rectum needs to be cleaned, so performing a quick enema on-site prior to the procedure is adequate and also more comfortable for the patient.

### Rectal biopsy procedure

Superficial 4–6 rectal mucosa specimens (2–4 mm diameter-Ø) were obtained by colon forceps with visual examination (Figure 1), avoiding the risk of bleeding, perforation or tissue damage, and immediately stored in ice-cold RPMI1640 with 5% (v/v) Fetal Bovine Serum (FBS). The procedure was performed in 3–15 min by experienced paediatric gastro-endoscopists assisted by a fellow (Additional file 2: Rectal biopsy procedure, Additional file 3: video S1 - Rectal biopsy procedure). We aimed to compare different biopsy forceps, as suction biopsies are not in current use at our endoscopy unit. In a first stage, we used the Olympus® 2.5 mm forceps (standard-oval without needle; required work channel size: 2.8 mm Ø; Shinjuku, Tokyo, Japan) and in a second stage the Endoflex® 3.4 mm (jumbo-oval without needle; required work channel size: 3.7 mm Ø; Voerde, Germany) in order to obtain larger specimens, optimizing the adequacy of the specimens for functional and biochemical studies.

### Sedation

From the 132 individuals enrolled in this study, 63 performed endoscopic procedures under sedation and 69 without. Intravenous sedation with midazolam (associated or not with meperidine) was performed for all individuals undergoing colonoscopy (n = 28) for other reasons than the rectal biopsy procedure (these were all non-CF individuals).

Sigmoidoscopy was done with or without sedation depending if the individual was already performing other procedures (like gastrostomy or upper endoscopy, n = 10) or depending on individuals’ will, collaboration or anxiety (n = 25, i.e., only 24% of the patients undergoing sigmoidoscopy preferred to be sedated). For children under 9 years old, the parents together with the paediatric gastro-endoscopists decided whether it was preferable to have sedation; while the individuals above 10 years old could choose to have sedation or not by themselves, because they had already a better understanding of the overall procedure. For those 35 individuals, sedation was performed as follows: a) intravenous sedation with midazolam (n = 9); b) intravenous anaesthesia with propofol (n = 2); c) intravenous anaesthesia with propofol + alfentanil (n = 5); d) sevoflurane (with nitrous oxide 1:1) inhalation (n = 8); and e) intravenous anaesthesia with propofol + sevoflurane inhalation (n = 11). In general, individuals were monitored for blood pressure, pulse, oxygen saturation and ECG tracing by an anaesthologist. Children could also choose if they wanted their parents with them during the procedure or not.

### Macroscopic evaluation

Prior to mounting in Ussing chamber, tissues were macroscopically evaluated regarding bleeding, mucus, biopsy thickness (presence of submucosa or not), and also for friability (tissue breakdown from manipulation), in a scale from 0 to 3 (from absence to clear presence of the descriptor), by two different technicians who were blinded for the individual condition (CF/non-CF), bowel preparation method and biopsy forceps used (Figure 1). Regarding tissue integrity resulting from the biopsing procedure, the scale was inverted to facilitate the technicians’ evaluation, i.e., 0 means full integrity of the specimen while 3 means that the tissue is fully disrupted (i.e. integrity is not present). Data on specimens’ macroscopic evaluation were obtained for 107 individuals and was averaged for 2–5 biopsies/individual to obtain a single value per individual, which was used for the correlations and tables presented here.

### Histology preparations

One out of the 4–6 rectal specimens collected per individual was fixed in 4% formaldehyde, embedded in paraffin and cut in thin sections (2–3 μM) for histological observation and data were obtained for 78 individuals. Sections were then deparaffinized dehydrated in xylene (twice, 10 min), re-hydrated with absolute ethanol (twice, 5 min), 95% ethanol (2 min), 70% ethanol (2) min and briefly rinsed with distilled water and stained with hematoxylin-eosin (HE) and Tricome’s Masson as previously [26]. Slides were mounted with xylene-based mounting medium. Again, pathologists assessed specimens blindly as above and data was obtained for 78 individuals.

### Ussing chamber measurements

Tissues were equilibrated in the micro-Ussing chambers for 30 min in perfused Ringer solution (in mmol/L: NaCl 145, KH_2_PO_4_ 0.4, K_2_HPO_4_ 1.6, D-glucose 5, MgCl_2_ 1, Ca^2+^-gluconate 1.3, pH 7.40, at 37°C) as previously described [12-14]. Measurements of basal R_te_ were used to assess tissue viability, after appropriate correction for fluid resistance (Additional file 2: Ussing chamber measurements and mounting of the tissue.).

### Biochemical assays: immunoblotting and immunofluorescence

Western blots were probed with a mixture (1:2000 dilution) of two monoclonal anti-CFTR antibodies, M3A7 and MM13-4 (Chemicon®, Millipore, Darmstadt, Germany) recognizing distinct epitopes to increase sensitivity as CFTR has low expression levels in native tissues [27,28]. Total protein extract (70 μg) was applied on the gel (Additional file 2: Biochemical assays; immunoblotting and immunofluorescence). For immunofluorescence tissue cryo-sections were incubated with monoclonal anti-CFTR antibody 570 (CFF, USA) and secondary polyclonal Alexa 488 anti-mouse antibody (Invitrogen, Carlsbad, CA, USA).

### Questionnaire used for patients’ assessment of the rectal biopsy procedure

Individuals were approached by telephone by two researchers that did not have contact with the rectal biopsy procedure to evaluate the overall procedure from bowel preparation to collection of the biopsy (Additional file 1, Figure S1, Additional file 2: Questionnaire used for patients’ assessment of the rectal biopsy procedure). Data were obtained for 75 individuals divided into 4 age groups, namely (yrs): 0–9 (n = 21); 10–19 (n = 33); 20–29 (n = 7); ≥30 (n = 14). Most patients were children (median age = 13 yrs). For children under 9 years old, the parents were asked to answer the questions as these children are very young to fully understand the procedure; while the individuals above 10 years old gave the responses themselves. Individuals were asked to rate the discomfort in comparison to other procedures such as nasal potential difference (NPD), nasal brushing, spirometry, sweat test, bronchoscopy and blood collection. All subjects enquired had undergone at least blood draws and sweat-Cl- testing for comparison with rectal biopsy (answers reported as “not applicable” were referred to individuals that were not able to establish a clear comparison, because they did not remind very clearly these procedures). They were also asked to comment on how many times they would accept repeating the procedure (Additional file 1: Figure S1, Additional file 2: Questionnaire used for patients’ assessment of the rectal biopsy procedure).

### Statistics

Statistical analyses were performed with SPSS software (v.19; SPSS Inc, Chicago, IL, USA) and a *p* value < 0.05 was considered as statistically significant. Unless otherwise stated, data are shown as mean ± SEM (*n* = number of individuals studied). Pearson coefficients (r) were used to find correlations and partial correlations between descriptors for macroscopic evaluation and R_te_ of biopsies. ANOVA, with Bonferroni *post hoc* correction when appropriated, was used to find differences between groups’ means with a 90% confidence interval. For Crosstabs regarding patients questionnaires, Pearson’s Chi-Square tests were used to determinate independence among variables analysed. Monte Carlo estimates of the exact *p*-value are provided whenever the data are too sparse or unbalanced for the asymptotic results to be reliable.

## Results

A flow-chart summarizing the technical biopsing aspects assessed in the present study is shown in Figure 1.

### Bowel preparation and biopsy forceps

The bowel cleaning procedures, consisting in administration of an oral laxative (for colonoscopy) or by enema (for sigmoidoscopy) allowed equally good visualization of the rectal mucosa and forceps during the sigmoidoscopy/colonoscopy procedure. Expectedly, jumbo biopsy forceps (3.4 mm Ø) generated larger specimens than standard forceps (2.5 mm Ø), thus facilitating the mounting of the tissue in Ussing chamber inserts.

Data referring to bowel preparation (Table 1, Additional file 1: Table S1) show that there are statistically significant differences (p = 0.054, 90% confidence interval) for tissue integrity between the preparations using NaCl 0.9% (1.19 ± 0.12) and glycerol 12% (1.82 ± 0.21). Oral mannitol was not statistically different from isotonic saline, independently of the biopsy forceps used (Table 1).

**Table 1:** **Summary of macroscopic evaluation data and bioelectrical measurements (R**_**te**_**) of rectal biopsies vs. bowel preparation and biopsy forceps**

**Bowel Preparation**		**NaCl 0.9%**		**Glycerol 12%**		**Mannitol 20%**	
**Biopsy Forceps**		**Jumbo**	**Standard**	**Jumbo**	**Standard**	**Jumbo**	**Standard**
**Tissue integrity**		1.00 ± 0.11	2.00 ± 0.35	1.48 ± 0.31	2.21 ± 0.23	1.02 ± 0.02	1.97 ± 0.30
**Friability**		1.23 ± 0.12	1.36 ± 0.24	1.50 ± 0.31	1.81 ± 0.22	1.13 ± 0.33	1.33 ± 0.25
**Bleeding**		1.01 ± 0.08	1.21 ± 0.18	1.27 ± 0.21	1.16 ± 0.29	1.05 ± 0.22	0.83 ± 0.32
**Mucus**		1.12 ± 0.11	1.18 ± 0.17	0.91 ± 0.24	0.88 ± 0.13	0.74 ± 0.13	1.06 ± 0.13
**Sub-mucosa (n)**	Yes	16	3	3	1	2	1
	No	42	9	4	4	12	10
**R**_**te **_**(Ω.cm**^**2**^**)**		21.82 ± 1.03	14.37 ± 1.21	18.75 ± 2.66	13.57 ± 2.70	18.69 ± 0.98	13.32 ± 1.11

NOTE: Results are mean ± SEM; n = 107.

Regarding biopsy forceps, mean values for tissue integrity (Jumbo = 1.07 ± 0.09 *vs* Standard = 2.04 ± 0.18) and also for R_te_ (Jumbo = 20.97 ± 0.81 Ω.cm^2^*vs* Standard = 14.01 ± 0.86 Ω.cm^2^) are statistically different between the forceps used (p = 5.51x10^-7^ for tissue integrity and p = 8.42x10^-8^ for R_te_, Table 1, Additional file 1: Table S2). Friability (i.e. tissue susceptibility to breakdown with manipulation) is the only parameter significantly affected by the presence of sub-mucosa (Additional file 1: Table S3, p = 0.008), but this may result from more tissue manipulation being required to remove the sub-mucosa. Regarding bleeding and mucus, their presence/abundance were not influenced by bowel preparation nor biopsy forceps (Table 1).

**Figure 2:**
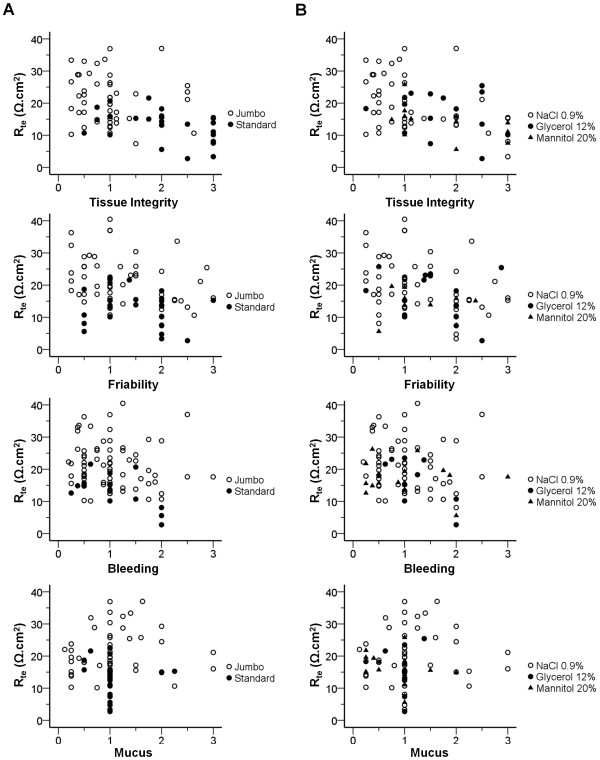
**Correlations between tissue transepithelial resistance (R**_**te**_**) and macroscopic descriptors (tissue integrity, friability, bleeding and mucus) according to A) biopsy forceps and B) bowel preparation (n = 107 individuals).**

In addition, we also found statistically significant correlations (Table 2, Figure 2) between R_te_ and tissue integrity (*r* = −0.438, p = 8.51x10^-5^) and between R_te_ and friability (*r* = −0.305, p = 6.65x10^-3^), which is supported by partial correlations with bowel preparation and biopsy forceps. Data concerning presence/abundance of blood and mucus do not correlate with tissue viability-R_te_ (Table 2, Figure 2). We found no influence by usage of sedation in the procedure regarding tissue viability (Additional file 1: Table S4).

**Table 2:** **Summary of the correlations and partial correlations (by bowel preparation and biopsy forceps) between tissue transepithelial resistance (R**_**te**_**) and macroscopic evaluation of biopsies**

	**Transepithelial resistance (R**_**te**_**)**	
	**Pearson ( *r*)**	**p-value**
**Tissue integrity**	**−0.438**	**8.51 x 10**^**-5**^
*Bowel preparation*	**−0.403**	**3.74 x 10**^**-4**^
*Biopsy forceps*	**−0.244**	**0.036**
**Friability**	**−0.305**	**6.65 x 10**^**-3**^
*Bowel preparation*	**−0.301**	**7.88 x 10**^**-3**^
*Biopsy forceps*	**−0.277**	**0.015**
**Bleeding**	−0.176	0.110
*Bowel preparation*	−0.185	0.093
*Biopsy forceps*	−0.182	0.100
**Mucus**	+0.078	0.507
*Bowel preparation*	+0.032	0.787
*Biopsy forceps*	+0.115	0.330

NOTE: Pearson (*r*) and p-values indicating significant correlations are highlighted (n = 107).

### Histological and macroscopic evaluation of rectal tissues and biochemical analysis

**Figure 3:**
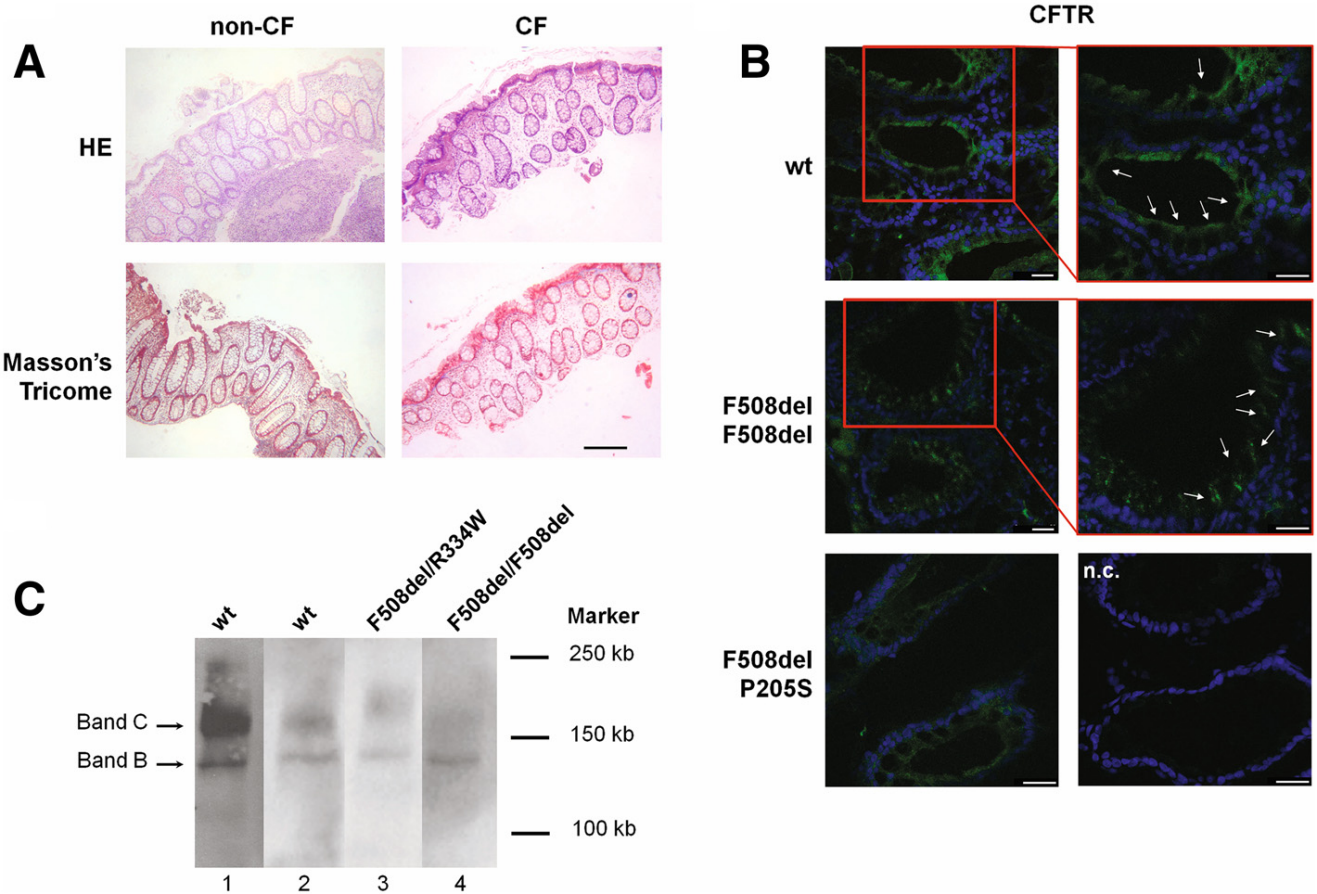
**Histological and macroscopic evaluation of rectal tissues and biochemical analysis. A**) Rectal biopsies (longitudinal cuts) were histologically evaluated by Hematoxilin-Eosin (HE) and Masson’s Tricome stainings in non-CF and CF tissues. Images show healthy epithelia, with no fibrotic processes and only some inflammatory cells were detected. Images shown are representative of the total and correspond to biopsies from a non-CF individual (left) and a CF patient (right) performed with jumbo (3.4 mm Ø) forceps after bowel preparation with glycerol (non-CF) or isotonic saline (CF). In HE-stained sections (top), nuclei are stained in blue and cytoplasm in red. In Tricome’s Masson-stained sections (bottom) collagen (fibrotic biomarker) is stained in blue, nuclei in black, and muscle and cytoplasm in red. Black scale bar represents 250 μM. **B**) Immunohistofluorescence of rectal biopsies showing nuclei in blue (DAPI staining) and CFTR in green. Images evidence CFTR at the membrane in a non-CF tissue (top panels) and also, albeit weaker, in a biopsy from a CF patient with the F508del/P205S-CFTR genotype (bottom panels). In contrast, a biopsy from a F508del-homozygous CF patient evidences intracellular CFTR staining (middle panels). A negative control (no primary antibody n.c.) was also performed. Scale bar represents 25 μm. **C**) Western blot of a single rectal biopsy from non-CF individuals (wt-CFTR, lanes 1–2) and from a CF patient with F508del/R334W-CFTR genotype (lane 3) evidence the presence of both immature and mature forms of CFTR (bands **B** and **C**, respectively; and from a CF patient with the F508del/F508del-CFTR genotype (lane 4) evidencing only immature form (band **B**) which is characteristic of the endoplasmic reticulum (ER) and thus corroborating the traffic defect associated with this mutation.

Histological examination of rectal specimens revealed no obvious abnormalities and only some inflammatory cells were found, independently of both tissue origin (CF or non-CF) or bowel preparation (Figure 3A).

Tissues were evaluated for integrity, friability, bleeding, mucus, and thickness of biopsy (presence of sub-mucosa or not) under a dissecting microscope by two different technicians in blinded way (Table 1). As shown above, the scores obtained from this evaluation were correlated with bowel preparation, biopsy forceps and measurements of transepithelial resistance (R_te_) to assess tissue viability (Table 2).

We have also used these native colonic tissues to further look at maturation pattern and localization of wt- and mutant-CFTR protein, which can serve as a valuable tool to evaluate the effects of CFTR-modulators together with Ussing chamber measurements of CFTR-mediated Cl^-^ secretion. As shown in Figure 3B, we were able to detect CFTR protein at the plasma membrane for both wt-CFTR and F508del/P205S-CFTR (P205S is a class IV mutation), but F508del/F508del-CFTR was retained into cytoplasm, close to the basolateral membrane. These findings were in accordance with the maturation pattern of CFTR protein (Figure 3C), which was found to be fully-glycosylated (presence of band C) for wt-CFTR and another class IV CFTR-mutant (R334W), but failed to mature in the biopsy from a F508del/F508del patient.

### Patient safety and comfort with overall procedure

No major complications (perforation, haemorrhage) were reported following NaCl 0.9% bowel preparation or jumbo biopsy forceps (allowing larger and more viable rectal specimens, 353 biopsies performed in total), thus making this a safe procedure. There was only one CF patient complaining about abdominal pain who was observed for 4 h after the procedure, but had no other complications and recovered by then from such pain. We also report other minor complications that cannot be fully related to the sigmoidoscopy procedure, (see Discussion).

Patients were asked by telephone to assess the rectal biopsy procedure by posing several questions targeting several aspects of patient assessment (Figure S1). The questions were divided into 3 broad categories (Additional file 2: Questionnaire used for patients’ assessment of the rectal biopsy procedure): *i)* procedural pain/discomfort and sedation requirement (questions 1,2,3,5); *ii)* comparison with other clinical/diagnosis procedures (question 4); and *iii)* acceptance towards the possible introduction of this method as an outcome measure in clinical trials (question 7). In addition, there was also a question regarding preconceptual concerns or discomfort and pain associated a priori with this procedure (question 6).

Data collected on patients’ (dis)comfort show that 57/75 (76%) of the interviewed individuals did not report high levels of discomfort, independently of sedation or age, but shows statistically significant differences (p = 0.032) regarding gender (Table 3): as there were more female patients saying that the overall rectal biopsy procedure is “somewhat uncomfortable”. Nevertheless, the majority of both female (32/41) and male (25/34) reported low levels of discomfort. Also, the great majority of the individuals inquired (78.7%) reported that this is a painless procedure, regardless of sedation, age or gender (Table 3). Only 2 individuals assessed the procedure as “Very painful” (data not shown). Moreover the vast majority of the individuals (88%, 66/75) accepted repeating this procedure for at least once more (18.7% for 1 more time; 12% for 2 more times; 4% for 3 more times; and 53.3% for 4 more times), while only a minority (12%, 9/75) do not wish to repeat it (Table 3), also independently of sedation, age and gender.

**Table 3:** Evaluation of comfort, pain and future repetition of the rectal biopsy procedure assessed by patients by gender, age group and sedation (n = 75)

		**Comfort**			**Pain**		**Future procedure**				
		**Very uncomfortable (18)**	**Somewhat uncomfortable (27)**	**Not uncomfortable (30)**	**No (59)**	**Yes (16)**	**0 (9)**	**1 (14)**	**2 (9)**	**3 (3)**	**4 (40)**
**Gender**	**Female**	9	20	12	34	7	3	8	6	0	24
	**Male**	9	7	18	25	9	6	6	3	3	16
**Age Groups (years)**	**0 – 9**	7	3	11	15	6	3	2	2	2	12
	**10 – 19**	6	16	11	27	6	5	7	5	1	15
	**20 – 29**	2	3	2	6	1	0	2	1	0	4
	**≥ 30**	3	5	6	11	3	1	3	1	0	9
**Sedation**	**No**	10	15	12	28	9	6	7	3	1	20
	**Yes**	8	12	18	31	7	3	7	6	2	20

NOTE: Results are n = number of individuals. Statistically significant differences were found between classification of procedure overall comfort regarding gender (p = 0.032).

When asked to indicate which steps of the procedure they considered as the least/most uncomfortable (Table 4), data shows that “the monitoring” was considered by the highest percentage (89.3%) as “Not uncomfortable” (76%) or “Least uncomfortable” (13.3%), followed by “the biopsing” (70.7%) and “the bowel preparation” (70.7%), and finally “the sigmoidoscopy” (66.7%). For the individuals being sedated, “the sedation” step was also well-tolerated, as much as “the monitoring step” (Table 4). Furthermore, as somewhat expected, sedation significantly enhances the rate of “Not uncomfortable” responses regarding “the sigmoidoscopy” (p = 0.016) and “the biopsing” (p = 0.003) procedures. No differences were found regarding gender or age (Table 4).

**Table 4:** Evaluation of the overall rectal biopsy procedure (n = 75)

	**Not uncomfortable**	**Least uncomfortable**	**Most uncomfortable**	**n.a.**
**Monitoring**	57	10	6	2
**Bowel preparation**	27	26	20	2
**Sigmoidoscopy**	36	14	23	2
**Biopsy**	42	11	20	2
**Sedation**	28	4	5	38

NOTE: Results are n = number of individuals. Statistically significant differences were found between classification of comfort for sigmoidoscopy (p = 0.016) and biopsy (p = 0.003) regarding sedation. n.a. means not applicable.

Concerning the comparative assessment of the rectal biopsy procedure with other clinical/diagnosis examinations (Additional file 1: Table S5), most of the individuals classified the overall rectal biopsy procedure as being “more unpleasant” than sweat test (76%), followed by spirometry (64%) and blood collection (53.3%), regardless of gender, age or sedation. No conclusions could be drawn concerning NPD, nasal brushing or bronchoscopy, because more than 65% of the individuals had not experienced those procedures or were unable to establish a comparison.

Finally, when patients where asked about their preconceptual concerns regarding the procedure (question 6), the reported answers were similar: 46.7% for preconception/taboo and 41.3% for discomfort/pain. The remaining 12% did not answer (data not shown).

## Discussion

Measurements of CFTR function in rectal biopsy specimens have proven its value in the fine-diagnosis of patients with milder or “non-classical” forms of CF, in particular when sweat test results are equivocal or borderline and/or if CFTR-disease causing mutations are not readily identified by DNA mutation analysis [12,14,16]. This approach also serves as a sensitive test to predict the prognosis when rare CFTR mutations are not identified by standard screening tests [12,14,16]. Compared to the airways, the rectal epithelium is easily accessible at any age, expresses higher levels of CFTR, thus increasing robustness of the measurements and, as shown here (Figure 3-A), does not undergo major secondary tissue destruction/remodelling as those occurring in CF airways [13].

Moreover, this technique has even potential for a much wider scope, namely to monitor diseases, affecting epithelial ion transport [29-31]. Although these studies were so far only performed in mice, the results are highly promising for the clinic.

Because functional and biochemical data are not available for most rare mutations, these specimens can also be used to establish functional and biochemical correlations with rare CFTR genotypes (Figure 3B, 3C), as also reported in other studies [12,14,16,28,32,33]. Furthermore, this approach may be used as well to validate efficacy of novel CFTR-modulator compound/drugs directly on human native tissues for such rare mutations [21-23]. Indeed, rectal biopsies are already in use as outcome measures in clinical trials for other diseases [24,25] and for some a high number of biopsies (n = 28) per patient has been reported without complications [25]. The same can thus be translated into the CF field.

Since good tissue viability is critical for quantitative assessment of bioelectric responses our recommendation is that specimens are maintained in appropriate medium on ice until used in functional measurements, which should be performed immediately [13]. To minimize edge damage (and consequent liquid/electric leakage), tissues should be mounted under a dissecting microscope for optimal orientation of biopsy on the insert opening and to prevent tissue damage with excessive instrument manipulation.

The present study aimed to determine whether and how bowel preparation for sigmoidoscopy and choice of biopsy forceps influence tissue viability for subsequent laboratory analyses, in particular, bioelectric measurements. We compared two commonly used solutions for bowel preparation at our endoscopy unit, namely glycerol and isotonic saline enema (for sigmoidoscopy) as well as oral mannitol (for colonoscopy). Our data show that isotonic saline solution, which we speculate is less harmful for the mucosa, is superior to glycerol-based preparation, but there are no significant differences between NaCl-enema and oral mannitol. Indeed, differences were detected between mean values for the tissue integrity parameter between biopsies obtained after NaCl- and glycerol-based bowel preparations, the former showing better results (Tables 1 and Additional file 1: Table S1).

The current study only focuses on the biopsing forceps procedure, as this is the routine procedure at our endoscopy unit. Indeed, this is the approach that we regularly use because it uses an endoscope which allows direct visual inspection of the mucosa area to be biopsied during the procedure to avoid hitting on damaged tissue or any abnormal vascular structures. This reduces the hemorrhage risk for the patient and increases the safety profile of the procedure. Indeed, excessive bleeding, although never occurring in this study, would be rapidly identified and thus immediately managed. It also allows avoiding biopsing the same site twice (Additional file 3 video S1: Rectal biopsy procedure).

However, previous [12,13,15,17-19] and recent reports [14,16,34,35] indicate that biopsies obtained by suction can be similarly applied (albeit without visual contact) and major complications have equally not been reported. In fact, the values for transepithelial resistance found by ourselves [14] (14–21 Ω × cm^2^) are comparable with those found previously using similar (forceps) [12] - 24 Ω × cm^2^ - or even different (suction) techniques [36,37]: 27 Ω × cm^2^, and 24–39 Ω × cm^2^ (no mean values given). That approach is thus expected to be equally tolerated by the patients’ while also providing adequate specimens for electrophysiology studies. Here, we did not compare suction and forceps biopsy, but just the forceps size (standard *vs* jumbo forceps) in terms of how sample size might affect its viability and also regarding safety of the procedure. Overall, mean values for tissue integrity (jumbo = 1.07 ± 0.09 *vs* standard = 2.04 ± 0.18) and also for R_te_ (jumbo = 20.97 ± 0.81 Ω.cm^2^*vs* standard = 14.01 ± 0.86 Ω.cm^2^) were shown to be statistically different (p = 5.51x10^-7^ and p = 8.42x10^-8^, respectively, Tables 1, Additional file 1: Table S2), with jumbo forceps providing the best results.

Importantly, data for both tissue integrity and friability show good correlations (positive and negative, respectively) with tissue viability (assessed by R_te_ measurements) and are influenced by bowel preparation and biopsy forceps (Table 2, Figure 2). Our data also show that collection of superficial rectal biopsies with (jumbo/standard) forceps constitutes a safe procedure, as we observed no complications, similarly to what others previously reported for studies on Hirschsprung [38] or inflammatory bowel disease [39].

Data concerning presence/abundance of bleeding and mucus do not correlate with tissue viability (R_te_), but interestingly there is a trend positive correlation between mucus and R_te_ (*r* = +0.078), indicating that presence of mucus (i.e. probably resulting from a less “aggressive” bowel cleaning) could somehow serve to preserve tissue viability (Table 2 and Figure 2).

Therefore, performing bowel preparation with isotonic saline and obtaining the rectal biopsies with jumbo forceps are demonstrated to constitute the best combination for the procedure (Table 1) with the highest mean values for R_te_ (21.82 ± 1.03 Ω.cm^2^) and the best for tissue integrity (1.00 ± 0.11). On the other hand, usage of 12% glycerol enema and smaller (standard) forceps produce the less viable tissues, namely (Table 1): worst tissue integrity (2.21 ± 0.23) and R_te_ (13.57 ± 2.70 Ω.cm^2^), and highest friability (1.81 ± 0.22), thus rendering quantitative determination of CFTR-mediated Cl^-^ secretion less reliable. It is likely that usage of the glycerol-based enema, acting as an osmotic laxative and thus increasing the luminal volume, which stretches the mucosa, makes it more susceptible to friability and disruption, compromising tissue integrity. Alternative for bowel preparation in children could be sodium citrate + sodium lauryl sulfoacetate, together with glycerol + sorbitol (“Microlax®”) or dioctyl sulfosuccinic acid sodium salt + sorbitol (“Clyss-go®”), which we had previously experienced to yield viable specimens if done the day before sigmoidoscopy (data not shown), probably allowing rectal mucosa to recover from this enema procedure. However, as these procedures were not rigorously assessed, we cannot compare them we the ones used in the present study.

The easy access to the patients rectum and the low innervation of this area minimizing pain, makes this approach to be expectedly well tolerated [12,14]. Moreover, modern gastroenterology techniques and instruments currently applied in outcome measures for clinical trials have made this approach increasingly simpler and easier [25]. Indeed, in the present study, concerning only the sigmoidoscopy/rectal biopsy procedure, sedation was used primarily to reduce anxiety and ensure cooperation (only for 24% of the sigmoidoscopy/rectal biopsy cases sedation was preferred, see Methods), especially in small children that generally demonstrate less cooperation in any medical procedure In cooperative, non-anxious patients, our experience recommends that the procedure is performed with no anaesthesia.

Regarding safety of the patients, one CF patient who complained of abdominal pain (see Results) was the only patient with inappropriate bowel cleaning, where more insufflation had to be used in order to carry out the procedure. This patient also had a clinical history of surgical interventions for meconium ileus and colonic adhesions, which might be related to the abdominal pain. We also report minor complications that cannot be fully related to the sigmoidoscopy procedure: one patient who vomited (although his parents informed that this usually happens when he fasts) and another patient who underwent both upper endoscopy and sigmoidoscopy also reported pain, but it is likely that the former procedure was the source of pain.

Thus, the present study also shows that this procedure with jumbo forceps is safe to be applied from young children to adults (age range was 6 months to 52 years), which application is relevant also for other disorders as Hirschsprung or inflammatory bowel disease. Although we have no experience, it also expected that it can be safely applied to newborns, namely those identified in increasing numbers as asymptomatic CF patients by the recently implemented extensive newborn screening programs, merely based on elevated serum concentrations of immunoreactive trypsinogen (IRT). Indeed, these patients, posing new challenges to the CF diagnosis and prognosis are likely candidates to undergo this procedure to find evidence of CFTR (dys)function.

One of the major limitations usually related to patient surveys, is that we can only rely on the responses of the individuals who agree to undergo certain procedures. In this case, we are relying on the perspectives of the patients who agreed to participate on the sigmoidoscopy/rectal biopsy procedure, so there is a selection bias that leads to underscoring the fraction of individuals who do not tolerate this kind of procedure. Nevertheless, we can only rely on the responses of the individuals who agreed to participate, as those who have not undergone the procedure can only have preconceptions and not real experience to assess it. Importantly, patient enquiries demonstrate that for the majority of the individuals (76%) the rectal biopsy procedure is not associated with high levels of discomfort due to the short procedure time (max 15 min, Additional file 3 Video S1: Rectal biopsy procedure), regardless of sedation (Table 3) and no significant differences were found between the “control group” – with an already established CF diagnosis- and the “diagnostic group” (data not shown). Moreover, this shows to be also a relatively painless procedure, as 79% of the individuals did not report pain (Table 3). Nevertheless, “the sigmoidoscopy step” was associated with the highest level of discomfort (Table 4) as possibly expected, probably because of the preconceptions associated with this type of procedure. Accordingly, the individuals interviewed classified the rectal biopsy procedure as more unpleasant than sweat test, spirometry or blood collection (Additional file 1: Table S5). But if these individuals are required to repeat the biopsy procedure, despite some preconception concerns comprising prejudice and discomfort, the great majority (88%) would accept doing it for at least one more time, with 53.3% of the patients accepting to repeat it up to four more times (Table 3). Importantly, we also have to take into account, especially when a research procedure which is not routine is attempted, that adults above 20 years of age are more receptive to repeat the procedure than adolescents or children (as reported by their parents).

## Conclusions

In conclusion, results from the present study recommend that in the case of forceps biopsy, rectal biopsies should be obtained with jumbo biopsy forceps after bowel preparation with NaCl isotonic solution to obtain viable specimens for bioelectric measurements for CF studies. The procedure is safe and is well tolerated from the patients’ perspective, demonstrating its feasibility as an outcome measure in clinical trials.

## Abbreviations

CF: Cystic Fibrosis; CFTR: CF transmembrane conductance regulator; Cl-: Chloride; HE: Hematoxylin-Eosin; FBS: Fetal Bovine Serum; IRT: Immunoreactive trypsinogen; Rte: Transepithelial resistance; NPD: Nasal Potential Difference; SOP: Standardized Operating Procedure; Ø: Diameter.

## Competing interests

Authors declare having no conflict of interests.

## Authors’ contributions

MFS, MS, AFR, JDR and MDA designed and conducted the study; MFS, MS, AMV, SRC, MAR, LM and RC collected data; MFS and MS analyzed data; MFS, MS, KK and MDA interpreted data; MFS, MS and MDA wrote the article; KK and JDR revised the article; AFR and MDA obtained funding. All authors read and approved the final manuscript.

## Pre-publication history

The pre-publication history for this paper can be accessed here:

http://www.biomedcentral.com/1471-230X/13/91/prepub

## Supplementary Material

Additional file 1: Table S1 Summary of macroscopic evaluation data and bioelectrical measurements. (Rte) of rectal biopsies vs. bowel preparation. **Table S2** – Summary of macroscopic evaluation data and bioelectrical measurements (Rte) of rectal biopsies vs. biopsy forceps. **Table S3** – Summary of macroscopic evaluation data and bioelectrical measurements (Rte) of rectal biopsies vs. presence of sub-mucosa. **Table S4** – Summary of macroscopic evaluation data and bioelectrical measurements (Rte) of rectal biopsies vs. sedation. **Table S5** – Comparison of the rectal biopsy procedure with other clinical/diagnosis procedures (n = 75). 6 **Table S6** – CF patients and CFTR genotypes (n = 67). **Figure S1** Rectal biopsy procedure patient assessment questionaire. Click here for file

Additional file 2 Supplementary Methods. Click here for file

Additional file 3: Video S1 Rectal biopsy procedure. Click here for file
