# Supplementary material for: Rectal forceps biopsy procedure in cystic fibrosis: technical aspects and patients perspective for clinical trials feasibility
Source: BMC Gastroenterol. 2013 May 20;13:91. doi: 10.1186/1471-230X-13-91 (PMC3679995; doi:10.1186/1471-230X-13-91)
Supplement: Additional file 1: Table S1 — Summary of macroscopic evaluation data and bioelectrical measurements. (Rte) of rectal biopsies vs. bowel preparation. Table S2 – Summary of macroscopic evaluation data and bioelectrical measurements (Rte) of rectal biopsies vs. biopsy forceps. Table S3 – Summary of macroscopic evaluation data and bioelectrical measurements (Rte) of rectal biopsies vs. presence of sub-mucosa. Table S4 – Summary of macroscopic evaluation data and bioelectrical measurements (Rte) of rectal biopsies vs. sedation. Table S5 – Comparison of the rectal biopsy procedure with other clinical/diagnosis procedures (n = 75). 6 Table S6 – CF patients and CFTR genotypes (n = 67). Figure S1 Rectal biopsy procedure patient assessment questionaire. [file 1471-230X-13-91-S1.pdf]

Table S1 – Summary of macroscopic evaluation data and bioelectrical measurements (R_{te}) of rectal biopsies *vs*. bowel preparation.

	NaCl 0.9%	Glycerol 12%	Mannitol 20%
**Tissue integrity**	1.19 ± 0.12	1.82 ±0.21	1.55 ±0.21
**Friability**	1.26 ± 0.10	1.65 ± 0.19	1.24 ± 0.19
**Bleeding**	1.04 ± 0.07	1.22 ± 0.17	0.99 ± 0.18
**Mucus**	1.13 ± 0.09	0.89 ± 0.14	0.88 ± 0.10
**R_{te} ($\Omega.cm^2$)**	19.12 ± 0.87	16.36 ± 1.96	16.20 ± 0.89

NOTE: Results are mean ± SEM; n = 107. ANOVA analyses indicates statistical differences between distribution of among the three groups for bowel preparation (p=0.039). Bonferroni post hoc test shows that difference is between NaCl 0.9% and Glycerol 12% (p=0.054; 90% confidence interval).

Table S2 – Summary of macroscopic evaluation data and bioelectrical measurements (R_{te}) of rectal biopsies *vs*. biopsy forceps.

	**Jumbo**	**Standard**
**Tissue integrity**	1.07 ± 0.09	2.04 ±0.18
**Friability**	1.26 ± 0.10	1.47 ± 0.15
**Bleeding**	1.04 ± 0.07	1.08 ± 0.14
**Mucus**	1.02 ± 0.08	1.09 ± 0.09
**R_{te} (Ω.cm^2)**	20.97 ± 0.81	14.01 ± 0.86

NOTE: Results are mean ± SEM; n=107. There are significant differences in tissue integrity ($p=5.51x10^{-7}$) and also between means of R_{te} ($p=8.42x10^{-8}$) for biopsy forceps.

Table S3 – Summary of macroscopic evaluation data and bioelectrical measurements (R_{te}) of rectal biopsies *vs*. presence of sub-mucosa.

	**Sub-mucosa presence**	
	**Yes**	**No**
**Tissue integrity**	1.49 ± 0.16	1.32 ±0.12
**Friability**	1.65 ± 0.18	1.18 ± 0.09
**Bleeding**	1.15 ± 0.14	1.01 ± 0.07
**Mucus**	1.20 ± 0.16	0.97 ± 0.06
**R_{te} ($\Omega.cm^2$)**	20.11 ± 1.45	18.95 ± 0.91

NOTE: Results are mean ± SEM; n = 107. Statistically significant differences were found between mean of friability regarding the presence of sub-mucosa (p=0.008).

Table S4 – Summary of macroscopic evaluation data and bioelectrical measurements (R_{te}) of rectal biopsies *vs*. sedation.

		Sedation	
		**Yes**	**No**
**Tissue integrity**		1.39 ± 0.15	1.34 ±0.13
**Friability**		1.22 ± 0.11	1.37 ± 0.12
**Bleeding**		0.97 ± 0.11	1.08 ± 0.08
**Mucus**		0.95 ± 0.08	1.13 ± 0.11
**Sub-mucosa (n)**	Yes	8	18
	No	42	39
**R_{te} ($\Omega.cm^2$)**		17.60 ± 1.05	18.85 ± 0.85

NOTE: Results are mean ± SEM; n = 107. No statistically significant differences were found between means of macroscopic and bioelectrical measurements regarding sedation.

Table S5 – Comparison of the rectal biopsy procedure with other clinical / diagnosis procedures (n=75).

	Less unpleasant	More unpleasant	n.a.
**Nasal Potential Difference**	9	11	55
**Nasal Brushing**	12	14	49
**Spirometry**	12	48	15
**Sweat test**	10	57	8
**Bronchoscopy**	3	7	65
**Blood collection**	25	40	10

NOTE: Results are n = number of individuals. n.a. means not applicable.

Table S6 – CF patients and CFTR genotypes (n=67).

CF Patients (n)	CFTR genotype
33	F508del/F508del
6	F508del/G542X
2	F508del/1716+18672 A>G
2	F508del/1717-1G>A
2	F508del/1812-1G>A
2	F508del/R334W
2	3120+1G>A/R1066C
1	F508del/2183AA>G
1	G542X/2183AA>G
1	F508del/3272-26A>G
1	A561E/A561E
1	F508del/G85E
1	G542X/I618T
1	W1282X/L206W
1	3120+1G>A/L206W
1	F508del/N1303K
1	F508del/P205S
1	G542X/P205S
1	F508del/R1066C
1	F508del/-
1	F508del/R1162X
1	R1162X/R1162X
1	G542X/R334W
1	F508del/R553X
1	F508del/S549R(T>G)

Figure S1

Rectal Biopsy Procedure Patient Assessment Questionnaire

Subject Name___________________________________ **Procedure Date:**_________
Gender: ____________ **Birth Date:** __________

1. Was it required to use sedation? **Yes / No**

2. Please classify the overall rectal biopsy procedure as:
A) Very uncomfortable B) Somewhat uncomfortable C) Not uncomfortable

3. Regarding the overall procedure, that includes:
i) the monitoring ii) the bowel preparation iii) the sigmoidoscopy iv) the biopsy v) the sedation (if used)

which one(s) did you consider to be:
A) Not uncomfortable B) The least uncomfortable C) The most uncomfortable

4. Regarding other clinical and/or research procedures that you have experienced, please classify the rectal biopsy procedure as being:
A) Less unpleasant or **B) More unpleasant**

than:
i) Nasal potential difference measurement
ii) Nasal brushing
iii) Spirometry
iv) Sweat test
v) Bronchoscopy
vi) Blood collection

5. Have you felt some pain during the biopsing procedure? **Yes / No**

In case of a positive response, please classify your pain as:
A) Very painful B) Acceptable Pain C) Almost unpainful

6. What bothered you more in this procedure: the *a priori* presumption that this would be an uncomfortable and painful procedure or the preconception and taboo of a rectal exam? ______________________________________

7. If the rectal biopsy was to be used as a future outcome measure in clinical trials, how many times do you think you would be willing to have it performed?
____ None
____ One time
____ Two times
____ Three times
____ Four times

8. Please express any additional comment you would like to provide us relative to this procedure.
____
