# Supplementary material for: Rectal forceps biopsy procedure in cystic fibrosis: technical aspects and patients perspective for clinical trials feasibility
Source: BMC Gastroenterol. 2013 May 20;13:91. doi: 10.1186/1471-230X-13-91 (PMC3679995; doi:10.1186/1471-230X-13-91)
Supplement: Additional file 2 — Supplementary Methods. [file 1471-230X-13-91-S2.pdf]

Supplementary Methods

Rectal biopsy procedure

When using jumbo forceps (Endoflex® 3.4mm; Voerde, Germany) a colonoscope with a working channel of 3.8mm Ø and an external diameter of 12.8mm (Fujifilm® EC-590ZWL; Tokyo, Japan) was used. For the standard forceps (Olympus® 2.5mm; Shinjuku, Tokyo, Japan) we used either a Olympus® (CV-VL model; Shinjuku, Tokyo, Japan) or a Pentax Ricoh® (FG-27X model, Tokyo, Japan) scope with a working channel of 2.8mm Ø and an external diameter of 9mm.

Individuals were examined in the left lateral decubitus position. The distal 10cm of the scope was lubricated with a water-soluble jelly and it was then inserted into the rectum by exerting gentle pressure by the scope tip on the anal sphincter until it relaxed. Insertion of the scope was done as quickly as possible, thereby limiting patient discomfort. Once the scope is the rectum (10 to 20 cm inserted), fluid that may be present was suctioned and the lumen was located and inspected by moving the tip of the scope. The normal rectal mucosa demonstrates a non-friable vascular system. Little or no air insufflation was used to avoid abdominal pain and bowel distension (more comfortable to the patient) and because it is easier to obtain superficial rectal biopsies. These were collected laterally at 15 to 25 cm from the anal verge in a low vascularized mucosa (Video S1).

Ussing chamber measurements and mounting of the tissue

To minimize edge damage, we mount tissues under a stereomicroscope allowing for optimal orientation of the small tissue specimen over the insert opening (circular aperture of 0.95 mm^2) and preventing tissue damage during manipulation with instruments, as before[1–3].

Luminal and basolateral surfaces of the epithelium were perfused continuously (5 mL/min). Typically, we perform experiments under open-circuit conditions, which resemble the *in vivo* situation more closely than short-circuit conditions. This approach may contribute to longer viability and a larger magnitude of drug responses[1–3]. To control for sample-to sample variability of *ex vivo* tissue specimens, we generally perform diagnostic bioelectric measurements on 2-5 biopsies per individual and data were averaged to obtain a single value for each individual. Transepithelial resistance (R_{te}) was determined by applying short (1 s) current pulses (0.5 µA) and the corresponding changes in transepithelial voltage (V_{te}) were recorded continuously. Values for the transepithelial voltage (V_{te}) were referred to the serosal side of the epithelium. The equivalent short-circuit current (I_{sc}) was calculated according to Ohm's law ($I_{sc} = V_{te}/R_{te}$).

Biochemical assays; immunoblotting and immunofluorescence

Rectal biopsy specimens from normal individuals and patients with established CF genotypes (liquid-nitrogen frozen after bioelectrical measurements and kept at -80ºC in homogenization buffer until being used) were homogenized in ice-cold 50 mmol/L Tris-HCl, pH 7.4, 150 mmol/L NaCl, 1mmol/L EDTA containing a protease inhibitor cocktail (Roche®, cidade) - Homogenization buffer. This material was then incubated 40 minutes with 1% (w/v) Nonidet P-40, 1% (w/v) sodium deoxycholate and 0.1% (w/v) sodium dodecyl sulphate (Lysis buffer). Insoluble material was removed by centrifugation and Laemmli buffer was added to supernatants. Protein extracts were quantified by modified micro-Lowry method and subjected to sodium dodecyl sulphate-polyacrylamide gel electrophoresis (7% acrylamide) separation[4]. Chemiluminescent detection was performed using Super Signal® West Pico Chemiluminescent Substrate (Pierce Rockford, IL, USA) or ChemiDoc™ XRS+ System with Image Lab™ Software system (Bio-rad, Hercules, CA, USA).

Thin sections (3-4 µm) of frozen rectal tissues (3-4 section replicates from each biopsy) were mounted on glass slides and stored at -80°C until immunofluorescence analyses. Briefly, sections were hydrated in phosphate-buffered saline for 5 minutes and fixed in methanol at -20ºC for 10 minutes[5]. After a blocking step (30 minutes in 1% (w/v) bovine serum albumin), sections were incubated with monoclonal anti-CFTR antibody 570 (Cystic Fibrosis Foundation, Bethesda, MA, USA) diluted 1:150 for 2h at room temperature[5] and after with Alexa 488-fluorescein labelled (Invitrogen, Carlsbad, CA, USA) diluted 1:300 for 1h, and mounted in Vectashield containing 4,6-diamidino-2-phenylindole (DAPI) to label nuclei (Vector Laboratories, Burlingame, CA, USA). Immunofluorescence staining was observed and recorded on a confocal microscope Leica TCS SPE (Leica®, Jehna, Germany).

Questionnaire used for patients' assessment of the rectal biopsy procedure

Although several attempts have been made to define patient satisfaction an acceptable definition is that it represents a patient's cognitive or emotional evaluation of a health−care provider's performance and is based on relevant aspects of a patient's experiences and perceptions[6]. Thus, different domains of assessment with gastrointestinal endoscopy procedures were described[7] and may include (i) the quality of care (endoscopy staff and environment); (ii) the comfort and tolerability of the procedure; (iii) the provision of an adequate explanation of the procedure; (iv) communication with the physicians before and after the procedure; and (v) waiting time or delays. Here, we focus on the comfort and tolerability of rectal biopsy procedure using a questionnaire covering: i) the choice of being or not sedated; ii) the evaluation of the discomfort of the overall procedure, including the monitoring, bowel preparation, rectoscopy, biopsing and sedation procedure; iii) comparison of the rectal biopsy procedure with other clinical and/or research exams such as nasal potential difference, nasal brushing, spirometry, sweat test, bronchoscopy and blood collection; iv)

classification of the pain deriving from the procedure; v) the concerns with this type of exam; and vi) the will to repeat the procedure if used as a research procedure (full questionnaire in Fig.S1).

This survey was applied to a total of 75 individuals: 60 CF patients and also 15 non-CF individuals. At the time of the survey, these 15 individuals were reported as CF-suspicious patients that had also undergone other common CF medical examinations, like sweat-test or spirometry, and thus included in this study. Only later, they were reported as non-CF individuals.

Chemicals and Compounds

All chemicals (highest available purity) were from Sigma-Aldrich® (St Louis, MI, USA) or Merck® (Darmstadt, Germany) except for culture media (GIBCO®/Invitrogen, Carlsbad, CA, USA).

References

1. Mall M, Hirtz S, Gonska T, Kunzelmann K. **Assessment of CFTR function in rectal biopsies for the diagnosis of cystic fibrosis.** *J Cyst Fibros* 2004, **3**(S2):165-169.

2. Hirtz S, Gonska T, Seydewitz HH, Thomas J, Greiner P, Kuehr J, et al. **CFTR Cl- channel function in native human colon correlates with the genotype and phenotype in cystic fibrosis.** *Gastroenterology* 2004, **127**(4):1085-1095.

3. Sousa M, Servidoni MF, Vinagre AM, Ramalho AS, Bonadia LC, Felício V, et al. **Measurements of CFTR-mediated Cl- Secretion in Human Rectal Biopsies Constitute a Robust Biomarker for Cystic Fibrosis Diagnosis and Prognosis.** *PloS one* 2012, **7**(10):e47708.

4. Farinha CM, Penque D, Roxo-Rosa M, Lukacs G, Dormer R, McPherson M, et al. **Biochemical methods to assess CFTR expression and membrane localization.** *J Cyst Fibros* 2004, **3**(Suppl 2):73-77.

5. Mendes F, Doucet L, Hinzpeter A, Férec C, Lipecka J, Fritsch J, et al. **Immunohistochemistry of CFTR in native tissues and primary epithelial cell cultures.** *J Cyst Fibros* 2004, **3**(Suppl 2):37-41.

6. Maciejewski M, Kawiecki J, Rockwood T. **Satisfaction in understanding health care outcomes research.** *Gaithersburg, Maryland: Aspen Publishers Inc* 1997, 67-89.

7. Yacavone RF, Locke GR 3rd, Gostout CJ, Rockwood TH, Thieling S, Zinsmeister AR. **Factors influencing patient satisfaction with GI endoscopy.** *Gastroint Endosc* 2001, **53**:703-710.
